# Transdiagnostic prevention in youth mental health, Part I: rationale, shared risk factors

**DOI:** 10.1038/s41386-025-02233-w

**Published:** 2025-10-02

**Authors:** Daphne J. Holt, Karmel W. Choi, Nicole R. DeTore, Oyenike Balogun

**Affiliations:** 1 Department of Psychiatry, Massachusetts General Hospital, Boston, MA USA; 2 Harvard Medical School, Boston, MA USA; 3 Department of Natural and Applied Sciences, Bentley University, Waltham, MA USA

**Keywords:** Stress and resilience, Risk factors

## Abstract

Over the past several decades, evidence has accumulated to support a transdiagnostic model of some of the processes underlying mental illnesses—that there are dimensional variations in genetic, environmental, neurobiological, and psychological factors that contribute to shared aspects of risk for developing psychiatric disorders, the majority of which emerge during childhood, adolescence and early adulthood. In this narrative review, the multiple, convergent lines of evidence for this understanding of psychiatric illness are summarized, and an integrated model of this evidence for both shared and non-shared risk factors and manifestations of psychiatric illnesses is proposed. This model can provide one testable framework for future investigations and a rationale for the development and dissemination of transdiagnostic approaches to mental illness prevention.

## Introduction

Although investments in the development and testing of new, more effective treatments for existing psychiatric illnesses will remain a priority of mental health research, intervening prior to illness onset is likely to be particularly cost-effective and have long-term benefits [1, 2]. A greater focus on prevention of psychiatric disorders may represent one important component of the overall effort to address the current mental health crisis in youth, with the worsening mismatch between the need for mental health care and its limited availability [3–5]. Psychiatric illnesses tend to emerge early in life, with 75% beginning before age 24 and 50% before age 14 [6, 7], with consistently high levels of psychiatric comorbidity over time [7] and variable responses to treatment [8, 9]. These and related statistics [3] highlight the need to allocate additional resources to early intervention and prevention efforts, which, over time, may reduce the incidence of these disabling conditions. Young people with psychiatric illnesses can accrue a lifetime of disability and financial burden related to the effects of chronic or intermittent symptoms, including the healthcare costs related to poor physical and mental health and missed opportunities for education, employment and the accumulation of wealth [10–15]. Thus, effective preventive interventions could dramatically impact the economic, medical and inter-generational costs of these conditions. However, although a wide range of potentially preventive approaches in psychiatry have been studied [16–19], little of this research has been translated, thus far, into “real world” practice.

There are likely a number of reasons for this, including the lack of consensus regarding objective markers of risk that can be used as screening and intervention targets, and the persistent stigma associated with mental health screening and care [20]. Also, the continued reliance on the Diagnostic and Statistical Manual (DSM) of Mental Disorders and related categorical models of psychiatric illness may represent another barrier to wider implementation of prevention approaches. This categorical approach is inconsistent with the large body of empirical evidence demonstrating that many DSM-defined diagnostic categories, which are considered to be useful clinically because they can be somewhat reliably assessed using established criteria, share clinical and biological features with each other. This pattern is most apparent in the high level of comorbidity within individuals, e.g., 75% of people with one psychiatric diagnosis have at least one other diagnosis [6, 7, 21]. In addition, the majority of DSM-defined categories are internally heterogeneous; people with the same diagnosis sometimes exhibit partially or entirely non-overlapping clusters of symptoms [22]. In fact, there is much evidence demonstrating that most forms of psychopathology are continuously expressed in the general population, from low to high levels of severity; thus the binary threshold required for DSM-defined diagnoses imposes a somewhat arbitrary distinction between illness and non-illness [21, 23, 24]. However, the continuous distribution of psychopathology in the general population provides an opportunity for implementing early detection and prevention strategies, given that symptomatic individuals who do not meet DSM criteria for a disorder may still benefit from regular monitoring or from a targeted intervention, since a subset will experience worsening symptoms and clinical progression [25–27]. These observations have inspired the recent development of staging models of mental health care, similar to those used in other areas of medicine, which provide tailored interventions for distinct levels of psychopathology and risk for progression [18].

Over the past several decades, some consensus has emerged supporting the view that DSM-defined disorders are unlikely to arise from unique, specific disruptions of a limited set of neurobiological mechanism(s) that lead to illness onset [21, 28, 29]. For example, the Research Domain Criteria (RDoC) approach [28], which has been employed by numerous researchers since its introduction almost two decades ago, is one manifestation of this consensus. Similarly, it has become increasingly clear that many (but not all) of the risk factors for psychiatric disorders identified by epidemiological [30–33] and genetic [34, 35] research are shared across multiple psychiatric conditions. Thus, screening for such transdiagnostic risk factors and targeting their manifestations (using transdiagnostic preventive interventions) could ultimately decrease the incidence of a wide range of conditions and impact a large portion of the population. Given that more than ~25% of the population will experience a psychiatric condition during their lifetime [6, 7], the benefits of widespread implementation of transdiagnostic preventive approaches could be substantial.

Based on this possibility, in this review, we summarize empirical evidence derived from a range of scientific domains, including genetics, epidemiology, neuroimaging, cognitive neuroscience, and psychopathology research, that support a *transdiagnostic prevention framework* for mental health. In addition, in a companion paper (Part II [36]), we describe examples of promising transdiagnostic preventive interventions that have been tested to date. Finally, in both articles, we discuss existing challenges and suggestions for future directions for the field.

## Evidence for shared *genetic* and *environmental* risk factors across psychiatric disorders

### Genetic risk

Some of the strongest evidence for shared risk factors across psychiatric disorders comes from large-scale studies of genetic risk factors for psychiatric illnesses. Genetics play a substantial role in risk across all psychiatric disorders, with family-based heritability estimates ranging from ~30% for major depressive disorder up to 60% and higher for schizophrenia [37]. Genetic liability for psychiatric disorders, as with all complex traits, is thought to arise from the accumulation of a large number (i.e., millions) of very small effects of common variations in the genome [38], with rare variation exerting larger effects in some cases [39, 40]. Notably, it has been known for some time that genetic liability for one psychiatric disorder is linked to a higher likelihood of developing a range of psychiatric disorders. For example, individuals at high genetic risk for major depression also have a higher risk of being diagnosed with bipolar disorder or a psychotic disorder [41] and vice-versa [42]. These findings align with those of earlier family studies showing that individuals with a first-degree relative with one psychiatric illness are at a higher risk of developing a psychiatric disorder, but not necessarily the same diagnosis as their family member [43, 44].

Thus, one of the most important findings of psychiatric genetics research over the past two decades is that there is a great deal of overlap in the genetic variants that confer increased risk for different disorders. This reflects the prevailing pattern of *pleiotropy*, which is widespread across the genome, in that roughly 90% of variants are estimated to be associated with multiple health outcomes [45]. Following the initial observation that many psychiatric disorders share high levels of overall genetic correlation [46], an early meta-analysis of eight psychiatric disorders identified 109 variants that were significantly associated with at least two disorders, and 23 that were associated with at least four [47]. Subsequent studies identified 152 genetic variants with pleiotropic effects across up to 11 different psychiatric disorders [48] and most recently, 268 loci across up to 14 disorders [49]. Many of the genetic variants with broad pleiotropy across multiple disorders play a role in early neurodevelopment—implicating genes that are initially expressed prenatally during the second trimester of gestation and persist into adulthood [47], including in radial glia and interneurons in the developing neocortex in mid-gestation [50].

It is important to note that there are different degrees of genetic overlap across psychiatric disorders. The multivariate genome-wide association study (GWAS) of eight psychiatric disorders found two dimensions of genetic risk with respect to mood versus psychotic psychopathology [51], while another identified further factor sub-groupings characterized by compulsive behaviors (e.g., Obsessive Compulsive Disorder (OCD), Anorexia Nervosa (AN)); mood and psychotic disturbances (e.g., Major Depressive Disorder (MDD), Bipolar Disorder (BD), and Schizophrenia (SCZ)); or neurodevelopmental conditions (e.g., Autism Spectrum Disorder (ASD), Attention Deficit Hyperactivity Disorder (ADHD), and Tourette’s Disorder). Some disorders were found to cut across these groupings, cross-loading on multiple factors, suggesting that these are not biologically distinct categories. More recent studies have expanded these factor sub-groupings of 11 disorders into four categories (neurodevelopmental, compulsive, psychotic, and internalizing), separating the mood (internalizing) and psychotic conditions. Most recently, meta-analytic work across 14 psychiatric disorders has identified five factor sub-groupings, replicating the neurodevelopmental, compulsive, and internalizing factors as well as identifying schizophrenia/bipolar and substance use factors [49]. However, despite these sub-groupings, there were still genetic signals identified as shared across all 14 disorders in this study, and these were linked to broad biological processes, such as synaptic functioning [51] or transcriptional regulation [49], suggesting the presence of fundamental mechanisms driving higher-order transdiagnostic risk.

In addition, although the focus of many of these studies has largely been on transdiagnostic risk for psychopathology, there are emerging studies that examine genetic factors underlying transdiagnostic resilience, i.e., the absence of a psychiatric disorder despite exposure to risk factors. For instance, a small GWAS conducted in Army soldiers [52] identified one genetic variant that was associated with the absence of multiple conditions—including major depressive disorder, generalized anxiety disorder, and post-traumatic stress disorder (PTSD)—following combat deployment, which was observed among a subset of individuals who had experienced high levels of combat stress exposure. The relationships between the genetics (and biology) of risk for and resilience to psychiatric illness remain unclear and is an active focus of current research.

### Environmental risk

Although genetic susceptibility plays a role in risk for psychiatric illnesses, it has also been well-established that a substantial portion of risk for these conditions is linked to environmental factors. Early research on this topic primarily measured the impact of the psychosocial environment on risk for psychiatric illness, including early childhood adversity (e.g., physical and emotional neglect, abuse), socioeconomic disadvantage, and family conflict. Other studies have focused on in utero exposures (nutritional deficiencies, toxins, infections) that impact later risk for psychiatric illness. More recently, researchers have also examined potentially detrimental characteristics of an individual’s physical environment, such as pollution, climate change, lack of green space, and urban living; this type of adversity has been estimated to account for about 20% of the attributable risk for mental illness overall [53]. Studies of the effects of substance use during adolescence have also identified transdiagnostic as well as potentially more diagnosis-specific effects (e.g., cannabis and psychosis) on risk for mental illnesses [54, 55]. Overall, convergent evidence has demonstrated that both the *timing* (e.g., prenatal, early, middle childhood or adolescence) [56, 57] and the *type* (e.g., toxins, trauma/social stress, features of the physical environment) of adversity can contribute to the impact of such exposures on the developing brain and mental health. Moreover, distinct types of adversities often act in combination, with complex interactions that can manifest variably across the lifespan. Lastly, an emerging body of research is also identifying protective factors that can mitigate the effects of early adversities.

Approximately half of all children will experience at least one form of adversity before they reach adulthood [58–60], and children exposed to adversity are about twice as likely to develop a psychiatric disorder compared to children who are not exposed to adversity, in a dose-dependent fashion, i.e., the risk is proportional to the amount of adversity experienced [60, 61]. For example, early epidemiological studies of childhood adversity, such as the well-known Adverse Childhood Experiences (ACE) study [62,] initially found graded relationships between ACEs and poor health outcomes, including ischemic heart disease. Importantly, the relationship between ACEs and ischemic heart disease was found to be more strongly mediated by the psychological variables (e.g., negative affect, anger) linked to experiences of childhood adversity than by typical health risk factors such as smoking or obesity [63]. Subsequently, a large body of research focused on the risk factors examined in the ACEs study has shown that childhood adversity increases the risk for developing a wide range of mental illnesses, including mood, anxiety, and psychotic disorders, personality disorders, and PTSD [64–68].

Investigations of the neurobiological mechanisms underlying these effects have suggested that different types of early childhood adversities may have distinct effects on the development of the brain and psychiatric outcomes, although these associations and their trajectories remain to be fully understood. Some of this evidence has come from animal models of early maltreatment [69] and from neuroimaging studies that have identified distinct changes in brain structure or function in individuals with histories of early traumatic experiences (such as abuse, exposure to violence) versus those who experienced early deprivation/neglect, when compared to those without any history of early adversity [70]. For example, these two types of early adversity (trauma vs. deprivation) may have opposite effects on amygdala volume [71]. Generally, diminished volumes of the amygdala, hippocampus and medial prefrontal cortex and over-responsivity of the amygdala, as well as changes in the function of the salience network (which includes the insula and dorsal anterior cingulate cortex, dACC), have been linked with experiences of early abuse, whereas changes in frontoparietal executive control circuits have been linked to experiences of early deprivation [70]. This two-dimensional model of early adversity is supported by evidence for related changes in emotional perception, learning, and regulation in trauma-exposed children, potentially reflecting a greater sensitivity to threatening information, and impairments in cognitive functioning linked with early experiences of deprivation [70]. However, this model requires further prospective confirmation.

In addition to studies of adversity occurring during early childhood, there have been investigations of the effects of a wide range of adversities occurring even earlier, during prenatal, in utero development - another important transdiagnostic environmental risk factor for psychiatric illness. For example, numerous studies have shown that nutritional deficiencies (due to famine) impact fetal development and later psychiatric outcomes. Large famines such as the Dutch Hunger Winter of 1944–45 and the Great Chinese Famine of 1959-61 have been linked to later increases in the incidence of schizophrenia [72–74] and depression [75, 76], with some evidence for associations between distinct gestational sensitive periods and vulnerability to different psychiatric conditions [31]. Similarly, the Helsinki Birth Cohort Study [77] found an association between slowed fetal growth and the later emergence of psychiatric illness over the lifespan. Lower weight/length and a smaller head circumference at birth, in various combinations, have been associated with the development of subclinical symptoms of ADHD and higher levels of internalizing and externalizing behavior in children [78, 79]. Also, extremely low gestational weight has been linked to the later onset of non-affective psychosis [74]. Similarly, poor diet and obesity in mothers during pregnancy have been shown to increase the risk for developing ASD, ADHD, anxiety, MDD, and SCZ in offspring [80–83], potentially due to hormonal or inflammatory dysregulation associated with poor nutrition [84].

In addition, studies have found that maternal exposure during pregnancy to teratogens and neurotoxins increase risk for psychiatric illness in offspring [85, 86], and large epidemiological studies [87, 88] have identified associations between maternal infections during pregnancy and the subsequent development in offspring of SCZ [89, 90], ASD [91, 92] and mood disorders [93], with evidence for a dose-response relationship between the number of infections and the level of risk for later mental illness [88]. There is also evidence that fetal exposure to maternal stress influences mental health outcomes of the child [94, 95]. These in utero exposures may exert their influence during early neurodevelopmental windows via effects on the development of stress response circuitry, impacting responsivity of the hypothalamic-pituitary-adrenal (HPA) axis and the peripheral autonomic nervous system by influencing the development of the brain circuitry regulating these systems [70, 96–98].

In addition to these proximal exposures or experiences of adversity during prenatal and early childhood sensitive periods, there are broader environmental stressors that can exert effects at multiple points during childhood, adolescence, and/or early adulthood. One well-studied category of this type of environmental stressor can be generally described as “social stress”. This type of stress includes early or ongoing experiences of bullying, social exclusion, or racial discrimination, which can increase the risk for developing a number of psychiatric disorders, including PTSD, mood, and psychotic disorders [99–103]. These associations may be mediated via dimensionally-expressed, transdiagnostic psychopathological traits or symptoms, i.e., internalizing and externalizing dimensions [100]. For example, a study of 5191 African American and Afro-Caribbean participants found that the relationship between perceived racial discrimination (across 9 situations, including unfair firings, denial of loans, subjection to inferior service and/or abuse by police) and the presence of psychiatric disorders was largely mediated by transdiagnostic internalizing and externalizing latent symptom factors [104], suggesting that the social stress associated with discrimination exacerbates vulnerabilities to broad dimensions of psychopathology. More recently, an emerging set of studies has focused on resilience promoters and adaptive functioning, particularly the role of strong relationships and social connections. For example, a large systematic review of racial discrimination and health outcomes found that, in 81% of the studies, aspects of social connectedness buffered the associations between the two [105].

Some studies of the biological effects of social stress have been conducted in both humans and animal models using the “social defeat” framework [106] –a construct that accounts for the feelings of loss, rejection, devaluation, and powerlessness that occur in the context of social hierarchies that expose individuals to chronic experiences of social exclusion. These types of experiences of social defeat represent a risk factor for a range of psychiatric disorders, including psychosis-spectrum disorders [107–109], anxiety, suicidality, PTSD [110], MDD, and substance use disorders [111]. There is evidence that social defeat-related stress is linked to sensitization of mesolimbic circuitry and diminished prefrontal control of amygdala and midbrain responses to stress [108, 112, 113], as well as dysfunction of midline cortical brain areas [114]. This type of stress sensitization of the brain may also occur in response to other environmental risk factors for psychopathology, such as childhood trauma and migration [107–109]. In addition, protective experiences, such as social support, may exert their beneficial effects via the same or related (modulatory) neural circuitry, counteracting the effects of maltreatment or social stress on brain function [115, 116].

Another category of environmental risk factors that, similar to social stress, may exert effects during multiple periods of neurodevelopment is those related to the physical environment. There is now a wide range of evidence suggesting that climate change, pollution, urban structural design, geopolitical events, and neighborhood disadvantage can influence mental health and brain development. For example, in utero exposure to air pollution has been linked to cortical thinning [117, 118], with specific regional effects depending on the type of pollutant and the timing of the exposure [53]. Neighborhood disadvantage has been linked to HPA axis dysregulation [119–121] and poor functioning of executive control circuits [122], as well as diminished brain volumes [123, 124]. Conversely, exposure to nature and green spaces appears to improve brain functioning, reducing neural responses to stress [125–127]. Overall, this body of research, although at a relatively early stage, has led to the recognition of the importance of measuring the effects of multiple different types of risk factors, the timing of the exposures, and the effects of cumulative exposures over time, rather than singular variables or events.

### Gene-environment correlations and interactions

Some transdiagnostic environmental risk factors, such as prenatal adversity [128], childhood adversity [129, 130] and social isolation [131, 132], may in fact have a partial genetic basis. Moreover, in some cases, the genetic liability associated with these environmental risk factors overlaps sizably with that of psychiatric disorders. For example, a GWAS conducted using the UK Biobank dataset [131] found that social isolation behavior was significantly genetically correlated with MDD (rg = 0.09), SCZ (rg = 0.10), and ASD (rg = 0.23), in addition to personality traits such as extraversion (rg = −0.44).

In addition, there is evidence that genetic variation may also modify how such environmental events contribute to the development of mental illness; through gene-environment interactions, an adverse environmental event may differentially impact an individual depending on their genetic vulnerability. For example, several studies have reported significant interactions between childhood trauma exposure and polygenic risk for MDD [133–135], although the direction of these interactions is less clear. It has been argued that interactions between genes and multi-factorial environments may best explain risk for developing mental illnesses [136]. Recent efforts to comprehensively model genome and environment -wide influences [137]—with environmental factors encompassing early life events, proximal (e.g., family, peer) and more distal (e.g., school) contexts—have identified genome-by-exposome interactions that account for substantial variance in internalizing symptoms in young people (an estimated 13%), above and beyond main environmental effects (16%). However, the sequence of processes that underlies many of these interactive relationships remains unclear.

While risks associated with adverse environmental events have been extensively studied, less is known about how positive environments may also interact with genomic variation to shape psychiatric outcomes. For example, early studies suggested that children with genetic risk factors associated with sensitivity to environmental effects may experience both more favorable mental health outcomes when they experience a positive childhood environment and less favorable outcomes within a negative environment [138]. This genetically influenced pattern of “differential susceptibility” (sensitivity to both negative and positive environments) has not yet been replicated in large studies. However, a similar pattern of results was observed in a recent study where adults with higher polygenic risk for MDD showed greater risk of MDD at lower levels of social support but reduced risk of MDD at higher levels of social support, across two cohorts [139]. In addition, a recent study [140] found that polygenic scores for “environmental sensitivity” specifically interacted with positive daily contexts to explain subsequent psychosis-spectrum symptoms. In light of these early findings, it is possible that certain interventions could also serve as positive “environmental exposures” that could interact with genetic risk factors.

## Potential *neural* manifestations of transdiagnostic genetic and environmental risk factors

### Changes in the brain associated with psychiatric conditions: limited evidence for diagnostic specificity

**Fig. 1:**
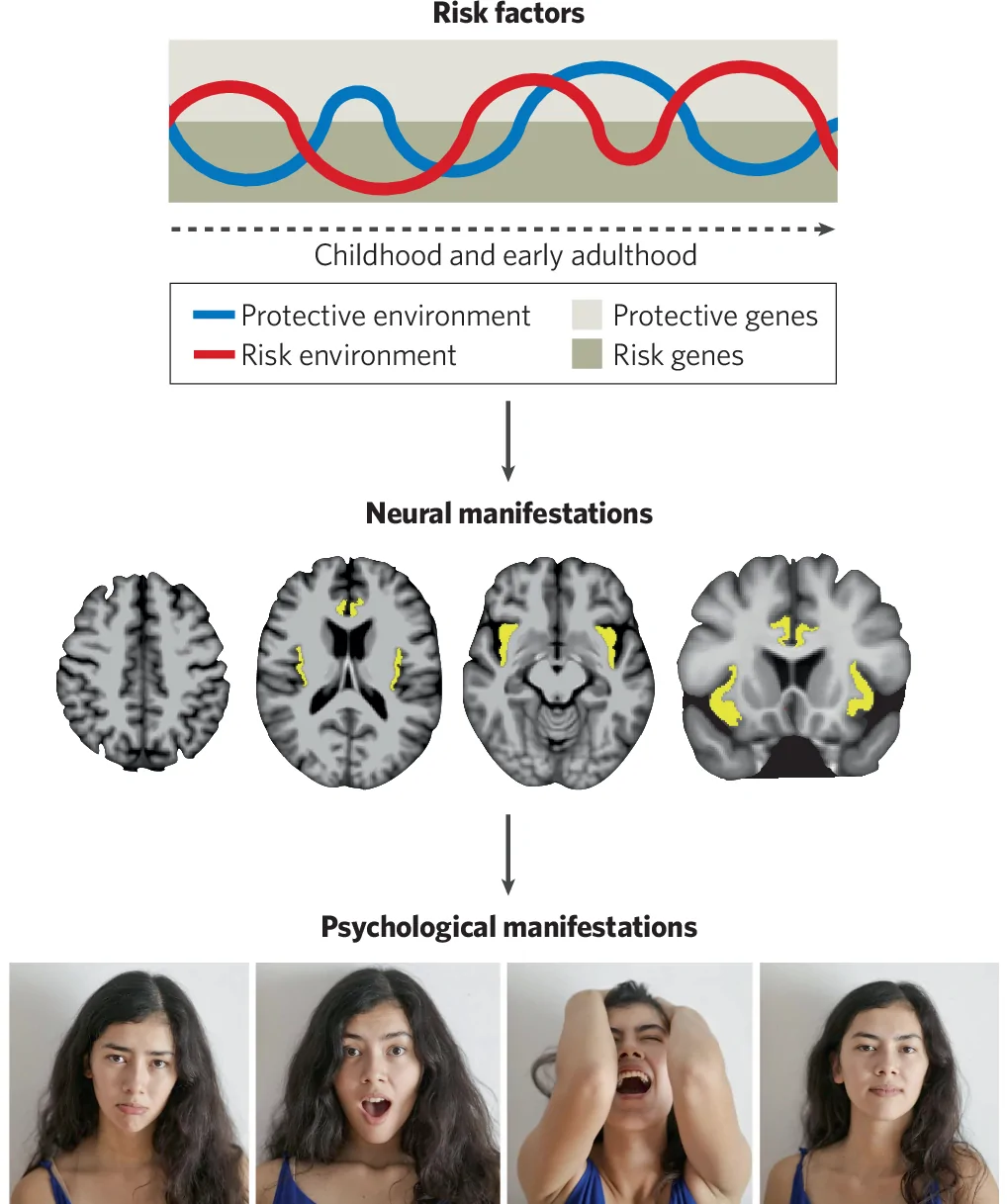
Schematic illustration of transdiagnostic risk factors and manifestations of psychopathology. This illustration, which schematically represents the interacting genetic and environmental risk factors and their neural and psychological expressions in an individual, outlines the overall organization of this review. In this article, we briefly summarize evidence for shared (transdiagnostic) and non-shared genetic and environmental risk factors, and potential neural and psychological manifestations of these risk factors, during the human lifespan. Omitted from this illustration (for the sake of simplicity) are the bi-directional interactions among these risk factors and phenotypes, e.g., influences of a person’s behavior on their environment and experience-dependent changes in brain structure and function.

Genetic and environmental risk factors for psychiatric disorders exert their effects by shaping brain development over the lifespan. Complex impacts of genetic risk factors and environmental influences on vulnerable brain structures can alter brain function in ways that ultimately give rise to sustained changes in psychological processes and symptoms of psychiatric disorders (see Fig. 1 for a schematic illustration of these relationships). Similar to research on genetic and environmental risk factors for psychiatric disorders, neuroimaging research in psychiatry has found surprisingly little evidence for diagnosis-specific neural features of DSM-defined disorders. One possible explanation for this is that this overall pattern of findings reflects, at least in part, current technical limitations of available brain imaging methods. Techniques with higher spatial or temporal resolution than those currently available, combined with the emerging application of artificial intelligence-based analytic approaches, may yield further advances and greater diagnostic specificity of findings. However, it is notable that the pattern of findings of psychiatric neuroimaging research to date general mirrors those emerging from psychiatric genetics and epidemiological studies (briefly summarized above), with a preponderance of shared or overlapping findings across psychiatric disorders.

Evidence for shared neurobiological features of psychiatric illnesses has primarily come from studies that use magnetic resonance imaging (MRI) based methods, as well as some using positron emission tomography (PET), to measure somewhat global features of the structure and function of the human brain. For decades, very few neuroimaging studies of psychiatric disorders focused on more than one diagnostic group; studies have typically compared patients diagnosed with one psychiatric disorder to a demographically-matched group of healthy participants without the disorder (a control group). However, even while employing this case-control design, similar or overlapping findings of studies of different disorders have been observed consistently. For example, structural and/or functional changes of the prefrontal cortex [141, 142], hippocampus [143–147], and amygdala [148–151] have been reported for mood, anxiety, and psychotic disorders. Some of these common patterns of findings across disorders can be explained by evidence for the role of certain neural systems in cognitive or emotional functions that are affected in multiple disorders, such as the evidence for the role of: (1) prefrontal and parietal cortices (frontoparietal circuitry) in cognitive control/executive processes [152–154]; (2) insula- dACC and medial temporal - ventromedial prefrontal cortical circuits (salience and limbic networks) in negative affect/internalizing symptoms [155–157] and (3) corticostriatal circuitry in reward-related functions [158, 159]. However, hypotheses regarding the possibility of common disruption of these processes and their associated circuitry across disorders still require confirmation in studies that use a dimensional, cross-disorder approach (e.g., an RDoC-based design) or combine datasets to compare disorders in meta-analyses (see below).

### Findings of cross-disorder meta-analyses of pooled MRI data

During the past decade, large meta-analyses, which pooled multiple datasets previously collected in studies of specific diagnostic groups, have enabled investigators to address questions about the potentially shared and unshared neural features of psychiatric disorders directly. The overall goal of these studies was to identify common and distinct changes in one or more neuroimaging outcome(s), such as gray matter density, volume, thickness, structural and functional connectivity measures, or functional activation, across multiple psychiatric disorders, relative to healthy control subjects. One of the first studies to use this approach conducted a meta-analysis of voxel-based morphometry (VBM) studies of six diagnostic groups (SCZ, BD, MDD, addiction, OCD, and anxiety), with a total sample size of 15,892 [160]. In this study, common gray matter reductions in the dACC and bilateral insula, the two primary regions of the salience network, were found across the diagnostic groups. This pattern of findings overlapped with those of a later, similarly designed meta-analysis of functional MRI studies of emotion processing [151], which reported evidence for aberrant functioning of the right insula (extending to the ventrolateral prefrontal cortex), ventromedial prefrontal cortex, thalamus, bilateral amygdala, hippocampus, parahippocampal gyri and inferior occipital cortex across the same six diagnostic groups studied in the earlier structural MRI meta-analysis [160].

Other studies investigating the loss of gray matter volume across disorders (which typically have included SCZ, BD and MDD cohorts, with the addition, in some studies, of groups with OCD, ADHD, ASD, PTSD and first degree relatives of psychotic patients) have identified shared/transdiagnostic gray matter reductions in the hippocampus [161, 162], parahippocampal gyrus and superior frontal gyrus [163] and increased volume of the putamen [164].

Similar to the transdiagnostic genetic studies, these neuroimaging meta-analyses have progressively increased in size over time, often with an increasing number of diagnostic groups included as well. A very large (N > 28 K) meta- and mega- analysis combining disorder-specific studies of the Enhancing Neuro Imaging Genetics through Meta Analysis (ENIGMA) consortium, which included structural MRI data collected from individuals with six disorders (MDD, BD, SCZ, OCD, ADHD and ASD), found that the regional effect sizes (for case vs. control structural differences) were highly correlated with each other for the MDD, BD, SCZ and OCD groups, but not for the ADHD and ASD cohorts [161]. These correlated effects could be represented as a shared latent factor (explaining 42.3-88.7% of the variance), which was expressed most prominently as diminished volumes of the hippocampus and fusiform gyrus.

Another study that used the same combined ENIGMA dataset examined the pattern of cortical thinning across disorders and found evidence for larger shared effects (case-control differences) in atrophy in transmodal (association) cortices and paralimbic regions than the effects observed in sensory and motor neocortical areas [165]. Moreover, this pattern was mirrored by the distribution of serotonin and dopamine receptors and transporters. The authors of this study highlighted that their findings aligned with the “structural model” of neuropsychiatric illness [166], which relies on evidence that specific characteristics of paralimbic cortical areas –- their limited laminar differentiation (i.e., a poorly developed or absent layer IV) and lower amounts of myelin, as well as their greater dendritic arborization and synaptic density –- are related to their greater plasticity (since their development occurs over a longer period of time) compared to neocortical areas. Consequently, these “high plasticity” paralimbic regions appear to be more vulnerable to injury than neocortical sensory and motor areas that have greater laminar differentiation (i.e., six layers) and high levels of myelin – protective features. This “structural model” of shared neural vulnerability across psychiatric disorders provides one testable framework that can be investigated further in longitudinal studies.

Other studies using the ENIGMA dataset, which have examined the predictive ability of normative patterns of (1) structural connectivity (typically determined by correlations between the thickness of cortical regions in healthy individuals) and (2) the regional patterns of distribution of molecular markers, including neurotransmitter profiles and gene expression (using PET data and detailed gene expression atlases of the human brain generated using postmortem material), have found evidence that both of these two types of information predict shared, cross-disorder abnormalities in brain structure. One study of 13 disorders [167], including neurodegenerative conditions such as temporal lobe epilepsy and Parkinson’s Disease, as well as SCZ, BD, ADHD, ASD, and MDD, found evidence that a combination of (1) high local/regional vulnerability, as determined by gradients of molecular markers, and (2) high levels of connectivity to other vulnerable regions, was most predictive of involvement in a cross-disorder pattern of cortical thinning.

Another analysis of a combined ENIGMA dataset also showed that the predominant pattern of shared reductions in cortical thickness across six psychiatric disorders, with major hubs in the temporal lobes and the ventrolateral prefrontal cortex, essentially followed the normal spatial pattern of functional connectivity of the human brain (as identified using the Human Connectome Project dataset) [168], suggesting that network level processes are involved in transdiagnostic vulnerability of the brain to psychiatric disorders.

This mounting evidence for the central role of “hub” neural circuits (those with high levels of inter-connectivity) in cross-disorder vulnerability was further supported by a DTI study of individuals with 8 psychiatric/behavioral conditions (SCZ, BD, ADHD, ASD, MDD, OCD, PTSD, and obesity) and 4 neurologic conditions, with a total of 1033 patients and 1154 disorder-matched controls [169]. In this study, multiple convergent analyses revealed that connections among regions with the highest levels of inter-connectivity, playing critical roles in the integration of information processing across the brain (including, unsurprisingly, some of the longest fiber tracts in the brain), showed the largest cross-disorder changes in connectivity. These findings remained, and were stronger in some analyses, when the data of the neurological patients were removed from the analyses, suggesting that this pattern is particularly characteristic of psychiatric disorders. The high metabolic demands of these central brain hubs, the physical length of their connections (and proportional vulnerability to white matter injury), and prolonged developmental time courses (with longer exposure to environmental insults) may render them particularly vulnerable to injury [169].

Further supporting the hypothesis that brain regions exhibiting a greater capacity for plasticity (with a longer developmental time course) are most affected in individuals who develop psychiatric illnesses is another ENIGMA dataset analysis [170] which identified shared patterns of cortical thickness across six psychiatric disorders and compared these to gradients of gene expression specific to pyramidal cells, astrocytes and microglia, using a virtual histology approach [171, 172]. The shared/transdiagnostic profile of cortical thickness differences across the 6 disorders was associated with the pyramidal cell gene expression distribution (explaining 56% of inter-regional variation); regions with the greatest cortical thinning showed the highest expression of these genes. Critically, many of the implicated genes were those involved in dendritic arborization, which is closely linked to cortical thickness and plasticity. Dendritic remodeling occurs in response to genetic influences and environmental stressors, and changes in dendrites have been observed in numerous psychiatric conditions [173–176]. Consistent with genetic association studies and studies of environmentally sensitive periods, these genes (which showed associations with the six disorders) could be categorized into two clusters: (1) a prenatal cluster enriched with genes involved in early neurodevelopmental processes, such as axonal guidance, and (2) a postnatal cluster enriched with genes involved in synaptic activity and plasticity.

### The “spreading pathology” hypothesis

One intriguing mechanistic model of these transdiagnostic neuroimaging findings is based on a hypothesis about the etiology of several neurodegenerative diseases, including Parkinson’s Disease and Alzheimer’s Disease, which suggests that these conditions begin with a relatively localized abnormality in one or more vulnerable, highly-connected hubs or “epicenters” in the brain, which is then transmitted over time to brain areas that are connected to that hub/epicenter [177–183]. It has been suggested that this type of propagation of a pathological process across circuits could occur in a number of different ways, e.g., under-activity of the hub region could lead to a loss of excitatory, inhibitory and/or trophic input to closely connected brain areas, which are then similarly affected over time, with functional and eventually structural, changes emerging in other closely, and then ultimately more distantly, connected areas. Alternatively, overactivity of the epicenter, leading to excitotoxic injury, could lead to other types of trans-synaptic changes over a network.

Evidence for these types of network-dependent effects has been found for a number of neurodegenerative disorders, suggesting that disease progression in these disorders could potentially arise from trans-synaptic transmission of a toxic cellular process, such as the misfolding and accumulation of certain proteins (e.g., tau, alpha-synuclein, huntingtin), leading to damage and eventual loss of synapses and neurons [177, 178]. A key feature of these models is that they typically incorporate both: 1) localized vulnerability factors (i.e., characteristics of the specific hub brain regions, such as their laminar organization and patterns of gene expression) and 2) features of network connectivity across the brain [167, 184]. Ongoing studies employing mouse models [185, 186], and testing computational predictions of disease progression [184, 187, 188], are attempting to identify the sequence of molecular processes and the degree to which common or disorder-specific cellular mechanisms may be involved in these network-level changes in neurodegenerative disorders.

However, only a few tests of this “spreading pathology” model have been conducted thus far in investigations of psychiatric disorders. One cross-sectional study of gray matter volume conducted in 534 individuals with psychotic disorders at different stages of illness (spanning first-episode to chronically psychotic patients) and healthy control subjects found evidence, using modeling techniques constrained by the architecture of the healthy human connectome, for a putative epicenter of pathology (volume reduction) in the anterior hippocampus that appeared to “spread” to other closely connected brain areas during illness progression [189]. These findings align with a large existing body of evidence for hippocampal abnormalities in psychotic disorders [146]; however, analyses of data collected in prospective, longitudinal studies [190, 191] are needed to further test this model.

Another preliminary finding that could be consistent with this spreading pathology hypothesis emerged from a study of individuals with a history of childhood maltreatment. Structural connectivity analyses of DTI data collected from participants with maltreatment histories revealed that those who had shown resilience (i.e., had experienced minimal symptoms of psychopathology) demonstrated lower efficiency (less integration of pathways across a network) of the networks of the amygdala and other nodes compared to those who had been exposed to maltreatment and develop symptoms. One interpretation of these findings, offered by the authors, is that limited long-range network connectivity of certain brain epicenters may have been protective for the resilient group, due to an associated reduction in transmission of a pathological functional or molecular process across closely connected brain regions [192].

In summary, the consistent findings that (1) brain regions that exhibit high levels of plasticity and are highly connected to other brain regions (hubs or epicenters) are disproportionately affected in neuropsychiatric disease, coupled with (2) the possibility that the underlying pathological process(es) involved in these disorders may originate in particularly vulnerable sites and then propagate across networks of regions closely connected to those sites, can be used to inform the design of future studies which rely on cognitive or symptom profiles, rather than diagnostic categories, to identify the neurobiological changes underlying psychopathology.

### Transdiagnostic functional connectivity studies

Other studies have examined transdiagnostic patterns of brain connectivity using analyses of resting-state fMRI data (acquired while the participant is “resting” (inactive) in the scanner). The results of many of these studies are not easily comparable to those of large meta-analyses of structural connectivity described above, given that they typically have sample sizes in the hundreds rather than the thousands and often focus on one or a few networks of the brain, such as the default mode network (DMN [193]), the “triple network” (the DMN, fronto-parietal network (FPN) and the salience network, (SN) [194]), or thalamocortical circuitry [195]. These studies have found evidence for a range of shared patterns of “dysconnectivity” across disorders in these networks. One study that measured cross-disorder changes in functional connectivity of the whole brain found that there was a shared pattern of lower “modularity” of networks across diagnoses, meaning that there was weaker differentiation among networks across the SCZ, BD, and MDD groups, compared to the healthy control sample [196]. Another whole-brain functional connectivity study that used a voxelwise multivariate regression method found evidence for shared changes in connectivity across SCZ, BD, and MDD samples (compared to control subjects) in the thalamus, cerebellum, sensorimotor cortices, and frontoparietal association cortices [197].

### Differences between diagnostic groups

Although many of the studies described above found prominent shared effects across diagnostic groups, there are several somewhat consistent diagnostic category-specific patterns of findings across some of these studies that are important to note. First, many of these studies have observed that the overall effect of having a psychiatric disorder (the transdiagnostic difference from healthy controls) was strongest in the SCZ group, followed by the BD group and then the MDD group (i.e., SCZ > BD > MDD > HC in magnitude of effects) [196–198], or that there were overall stronger effects in the psychotic group compared to the non-psychotic groups [142, 151]. The SCZ > BD > MDD pattern of effects has been observed in numerous studies using a wide range of data types, including resting-state functional connectivity [196, 197], DTI [198], low frequency fluctuations of resting-state fMRI data [199], and morphometric [200] data. For example, one study showed, using DTI data, that the “global efficiency” of whole brain connectivity showed a SCZ > BD > MDD pattern of effects, with the SCZ group showing the lowest efficiency; however, despite this pattern of results, machine learning analyses could not discriminate between any of the disorders, only discriminating each disorder from the healthy control group [198].

A related common pattern of results of the transdiagnostic neuroimaging investigations conducted to date is a finding of greater overlap in neural changes (cortical thinning or dysconnectivity) between the SCZ and BD groups compared to the extent of overlap of these two groups with the other diagnostic groups [165, 167, 201]. This pattern of findings is reminiscent of the pattern of shared effects observed in genetic risk [35, 49].

### Current limitations of this line of research and future directions

Lastly, several of these cross-disorder neuroimaging studies found greater changes in the brain, compared to controls, in those with greater illness severity [164], longer duration of illness [201], or earlier onset of illness [198], or in those who were at a later stage of illness [167]. These findings highlight one important limitation of this research thus far – that the majority of the large, multi-diagnosis neuroimaging data analyses conducted to date have been cross-sectional in design and conducted using data collected from adults with psychiatric diagnoses. Thus, some or many of the effects observed could be due to secondary effects of having the illnesses over time (including treatment effects), rather than to the underlying pathophysiological mechanisms causing the conditions. Also, some findings may be related to the *progression* of the underlying pathological processes. Disentangling the effects of secondary illness factors, illness worsening/ progression, and the etiological mechanisms contributing to illness onset may become increasingly possible as data from currently ongoing, large-scale, longitudinal studies (e.g., [190, 191]) become available.

## Potential *psychological* manifestations of transdiagnostic genetic and environmental risk factors

### Personality, temperament, and cognition

Genetic and experience-dependent variations in brain structure and function across individuals are ultimately manifested as subjective sensations, emotions, thoughts, and, ultimately, behaviors. Disruptions in the mechanisms underlying these processes, or mismatches between them and environmental contexts and expectations, can give rise to symptoms of psychopathology. It has been established that certain enduring or fluctuating patterns of perceptions and behaviors vary in magnitude continuously across the general population, and, at the extremes of this variation, some of these tendencies can be highly disabling. There is a long tradition in psychology research of measuring and categorizing these dimensions across general population samples (children and adults) in the study of personality, temperament and neurocognition.

Specifically, replicable personality dimensions have been identified, using the overlapping Big Five (extraversion, agreeableness, conscientiousness, neuroticism, and openness to experience) [202, 203] and Big Three (negative emotionality, positive emotionality and disinhibition) [204, 205] personality models, with their essentially equivalent constructs of 1) neuroticism and negative emotionality, and 2) extraversion and positive emotionality [206]. Studies of the links between these personality dimensions and psychopathology have consistently found associations between neuroticism (also sometimes referred to as trait negative affect) and many forms of psychopathology, including mood, anxiety, and psychotic disorders [206–211]. Similarly, low conscientiousness has been non-specifically linked to multiple forms of psychopathology [206], as well as to poor physical health [212, 213]. Moreover, factor analyses jointly modeling psychiatric and personality traits in a population-based sample of adults found evidence that all Big Five traits map onto one latent factor for psychopathology risk, characterized by high neuroticism and low conscientiousness and agreeableness [214], suggesting that dimensions of personality traits can be conceptualized as another transdiagnostic model of mental health. Aspects of these dimensions likely have a genetic basis; for example, neuroticism has been found to be highly heritable (up to 47%) in twin studies [215], with robust GWAS findings that support its potentially causal relationship with multiple psychiatric conditions [216].

A construct that is related to personality is temperament–-the individual differences in emotional reactivity and self-regulation that are evident very early in life and remain relatively stable over the lifespan. Behavioral inhibition is one temperament type observed in early childhood characterized by a tendency to exhibit fearfulness or avoidance in novel situations or with unfamiliar people [217]. Behavioral inhibition has been linked to an elevated risk for developing social anxiety during childhood and adolescence [217–219]. In addition, dimensional measures of behavioral inhibition and related traits are also elevated in people diagnosed with SCZ, BD, and MDD [220–222], suggesting that this trait represents another transdiagnostic risk factor for psychiatric illness that is closely linked (overlapping in underlying biology) to neuroticism and internalizing symptoms (i.e., depression and anxiety, see below).

A related trait—impulsivity—which may reflect the inverse of inhibition, has also been studied extensively in relation to risk for psychiatric disorders. Impulsivity has been positively correlated at a genetic level with both internalizing and externalizing disorders, and negatively with compulsive disorders, as well as with other transdiagnostic traits such as insomnia and cognitive ability [223].

In parallel with personality and temperament assessments, the fields of neuropsychology and cognitive neuroscience research have generated quantitative, dimensional norms of performance for every major domain of cognitive function, including executive function, episodic memory, language processing, and social cognition, among others. Cognitive impairment is common in SCZ, and to a lesser extent in BD and MDD [224–226], and having a higher IQ may be somewhat protective against developing SCZ in at-risk samples [227, 228]. Thus, cognition is another dimension of psychological functioning that is inter-related with personality and temperament.

Lastly, another category of traits that has been less well-studied to date are protective, resilience-enhancing traits or capacities [229], such as positive affect, optimism, social skills, compassion, self-awareness, emotion regulation capacity, and cognitive flexibility, which may reduce the risk of developing psychopathology [230–236]. It has been shown that some portion of these protective capacities is genetically determined (~40%) [237], and some can be modified and learned [238, 239]. In some cases, the effects of these protective characteristics may not merely represent the inverse of risk factors [240] but may have independent effects that interact with risk in complex (and thus far, poorly understood) ways.

### The Research Domain Criteria

The explosion of research in cognitive and affective neuroscience during the past half century, and the related effort to apply cognitive neuroscience concepts and methods to investigations of psychiatric disorders, gave rise to the development and implementation of a neuroscience-based transdiagnostic, dimensional model of psychopathology, the Research Domain Criteria (RDoC) framework [28, 241]. Similar to dimensional personality and psychopathology models, the RDoC framework has aimed to address barriers to progress presented by the poor correspondence between DSM-based diagnostic categories and the empirically-derived dimensional distribution of clinical, behavioral, and biological data across psychiatrically healthy and ill populations. RDoC focuses on specific, empirically-defined “constructs” (e.g., working memory, reward sensitivity, threat learning), which each represent an established dimension of behavior known to rely on a specific neural system. The constructs are intended to be studied across psychiatrically heterogeneous samples displaying a wide range of functioning. RDoC constructs are also grouped into broader domains of functioning (the Negative Valence Systems, Positive Valence Systems, Cognitive Systems, Social Processes, Arousal and Regulatory Systems, and Sensorimotor Systems), and RDoC-based studies tend to collect several types of data (“units of analysis”), including self-report, behavioral assessments, and neurophysiological measurements. Some RDoC-informed studies focus on a single psychological process or symptom (e.g., social anhedonia) or behavior (e.g., interpersonal distancing) and its neurophysiological correlates *within* a DSM-defined diagnostic category (e.g., SCZ [242, 243]), given the heterogeneity within samples of DSM-defined psychiatric disorders, whereas other studies are truly transdiagnostic or “a-diagnostic” [244].

It is also important to note a limitation of the RDoC approach that is similar to those associated with using DSM-defined illness categories in research. The RDoC constructs may or may not show consistent relationships with underlying neural mechanisms across neuropsychiatric disorders. For example, the construct/symptom of anhedonia appears to be somewhat heterogeneous in its expression both clinically and mechanistically within and across disorders [245]; shared and non-shared characteristics of reward processing have been observed across anhedonic individuals with diagnoses of MDD versus SCZ [246]. Thus, some of the RDoC constructs may not be more biologically uniform than DSM-defined categories. Ultimately, data-driven clusters [247] or dimensions (see below) of quantitatively-defined cognitive or behavioral processes that have clinical relevance may be most useful for mechanistic studies of psychiatric disorders.

### Dimensional models of psychopathology

Variations in symptoms of psychopathology, from subclinical to clinical levels, have been studied using similar approaches to those used in research on personality and temperament, and a number of models have been generated based on factor analyses of large psychopathology assessment datasets. An early model distinguished between two factors, internalizing and externalizing symptoms [248]. Later iterations of this model also included a psychotic experience or thought disorder factor [21, 249]. Subsequently, the high levels of correlation among the factors within these models, with the internalizing factor accounting for the largest amount of variance, led to the adoption of a hierarchical structure for these models, which could account for the strongly correlated relationships. The Hierarchical Taxonomy of Psychopathology (HiTOP) is the most well-developed version of this hierarchical approach [23]. HiTOP includes iterative subcategorizations (starting with larger internalizing, externalizing, thought disorder “spectra” and others), which are composed of increasingly narrow and specific constructs and finally, groupings of psychiatric disorders [250, 251].

A related line of psychopathology research has also identified, using factor analyses, a single common general psychopathology (“p”) factor [21], which is now typically incorporated into these hierarchical symptom models, placed at the highest level of the hierarchy (before it branches into the component factors). This unitary p factor is not well-understood in terms of what it represents psychologically, but it may be comprised of some of the most commonly shared aspects of psychopathology, such as neuroticism, emotional dysregulation, and cognitive impairment. The existence of some type of shared p or transdiagnostic factor may help explain the high rates of both concurrent and sequential comorbidity across all classes of psychiatric disorders [7, 21].

Based on these clinical observations, it has been proposed that there is a longitudinal, developmental progression of psychopathology in which a common “pluripotent” risk state or p factor present in many children is, in some instances, followed by worsening internalizing or externalizing symptoms and, subsequently, in a smaller vulnerable subset, further worsening, with the emergence of psychotic symptoms [21]. This type of progression model is consistent with data showing that the majority of individuals diagnosed with either SCZ or BD experience depression (or more broadly, affective or internalizing symptoms [252]) prior to the onset of those disorders [253–255]. In other words, since some episodes of depression are followed by the onset of specific symptoms of BD or SCZ (mania and/or psychosis), an episode of depression may represent, in some individuals, a risk state or an intermediate stage, on a trajectory towards the development of a more persistent and severe disorder [256].

Notably, this longitudinal progression model is consistent, in overall structure, with the pattern of many transdiagnostic neuroimaging findings described above, which suggest that there is a gradient in magnitude of brain abnormalities from non-psychotic to psychotic disorders (e.g., greater magnitude of effects in SCZ/BD compared to MDD). Furthermore, this longitudinal progression model also maps onto an emerging “staging” model of treatment, in which transdiagnostic interventions are provided at early stages, followed by increasingly symptom-specific interventions at later stages (when progression and greater clinical differentiation occurs) [18, 256].

Finally, these findings also align with genetic studies that have begun to examine the heritability of transdiagnostic risk for psychopathology, using these dimensional measures of shared variance in psychopathology. For example, twin studies modeling the p factor in youth across internalizing and externalizing symptoms have identified substantial heritability (50-60%) of p [257]. Related to this, other genetic studies have found that a measure of general cognitive ability, denoted as “g”, is associated with p, and these two transdiagnostic factors appear to be linked across development [258]. Thus, changes in cognitive and affective processes associated with psychopathology, and the frequently accompanying functional impairment, may arise from related neurobiological mechanisms.

### Subclinical symptoms

Consistent with the research on the dimensional models of psychopathology described above, it is now widely recognized that subsyndromal symptoms of specific psychiatric disorders, such as MDD and SCZ, are associated with elevated risk for developing a diverse range of psychiatric disorders, not just the “homotypic” disorder. For example, the presence of subclinical symptoms of MDD is associated with an increased risk for developing anxiety [259] and psychotic [260, 261] disorders, in addition to mood disorders [262–264]. Also, subclinical symptoms of psychosis, sometimes called “psychotic experiences” or “psychotic-like experiences”, are usually mild and transient, relatively common, and, in the majority of cases, are not followed by the onset of a psychotic disorder [265, 266]. However, it is also true that these subclinical psychotic symptoms are associated with an elevated risk (up to a 10-fold increase, depending on their severity) for developing a psychotic disorder [267–269] and/or a mood or anxiety disorder (4-fold and 3-fold increase in risk, respectively), as well as elevated risk for suicide attempts [270] and overall disability [271, 272]. Overall, adolescents presenting with psychotic experiences are significantly more likely to seek mental health services, receive any psychiatric diagnosis, and receive treatment with psychotropic medication than adolescents without these symptoms [273]. Thus, since these and other subclinical symptoms of psychopathology are some of the most easily assessed (given the widespread availability of brief, validated self-report scales that measure them) transdiagnostic risk factors for psychiatric disorders, they have been used more than any other risk factor as targets of early detection screening and prevention approaches (see the companion article of this review, Part II [36], for further details).

### Neuroimaging studies of dimensional aspects of psychopathology

Given the recent development and adoption of these different dimensional models of psychological/cognitive function and psychopathology (e.g., RDoC, HiTOP), increasingly neuroimaging studies are measuring associations between: (1) variation in brain structure or function and (2) variation in cognitive, affective, behavioral, or symptom dimensions. For example, one study of reward processing in individuals with genetic or clinical risk for psychosis, MDD, BD, or SCZ examined the relationship between whole-brain functional connectivity, using a voxelwise multiple regression approach, and self-reported reward responsiveness. The analyses revealed that higher reward responsiveness was linked to greater functional connectivity of the nucleus accumbens with components of the default mode network, as well as lower connectivity of the nucleus accumbens with cognitive control regions [274]. Another example of this type of dimensional approach is represented by the studies of neuromelanin-sensitive MRI signal of the midbrain [275] that have found associations between the intensity of this signal and dimensional levels of psychotic symptoms in a variety of psychosis-spectrum samples [276, 277].

Some studies have also started to examine associations between neural measures and data-driven transdiagnostic dimensions of psychopathology, such as the p factor. One study examined functional connectivity data of people with diagnoses of SCZ, schizoaffective disorder, BD, ADHD, and a healthy control group and found that three transdiagnostic latent factors, computed from 54 symptom and behavioral measures, that reflected general psychopathology, cognitive dysfunction, and impulsivity dimensions, were each strongly linked to the functional connectivity of sensory and motor areas [278]. Similarly, associations between levels of p (derived from a battery of psychiatric symptom assessments) and changes in white matter integrity of the pons, gray matter volumes of visual cortex and the cerebellum [279] and resting-state functional connectivity of visual association areas [280] have been observed, as well as links between levels of p and overall cortical thickness, particularly in heteromodal association cortices [281]. Lastly, a study conducted in a sample of ~1000 youth (the Philadelphia Neurodevelopmental Cohort) found that a general p factor was associated with elevated perfusion of the dACC and left rostral ACC, and reduced connectivity between the dACC and caudate nucleus [282], reminiscent of prior findings of transdiagnostic changes in gray matter volumes of the salience network [160]. Although the findings of these p factor/dimensional neuroimaging studies have been somewhat inconsistent to date, studies conducted in larger samples, ideally focused on objectively measured psychological or behavioral functions in addition to self-report symptom measures, may shed further light on dimensional relationships between characteristics of the brain and psychological functions.

## Summary and proposed model

Thus, to briefly summarize what we have reviewed above, there is convergent evidence for the existence of some shared genetic and environmental risk factors, as well as shared changes in brain and psychological functions, across psychiatric disorders, and for variation in neural and psychological processes across the general population that spans adaptive to disabling, pathological levels. Research is beginning to identify links between common transdiagnostic risk factors (genetic and environmental) and candidate phenotypes (neural, psychological, and clinical), with the causative mechanisms remaining to be discovered. In addition, there is also emerging evidence for some distinct (non-shared) risk factors, as well as syndromally-specific neural/psychological phenotypes that may emerge over time in some individuals.

Notably, recent studies of (1) shared genetic risk and (2) symptom clustering across disorders have generated models that partially align with each other. For example, a recent factor analysis of genetic risk associated with 14 psychiatric disorders produced a 5-factor solution (internalizing disorders, SCZ/BD, substance abuse disorders, neurodevelopmental disorders, and compulsive disorders factors) that could be modeled equally well (with an approximately equivalent fit) with or without inclusion of a common p (transdiagnostic) factor. The factors of this model based on genetic susceptibility are similar to those of the HiTOP model of psychopathology, which includes internalizing, externalizing, and thought disorder factors, and often a common p factor as well.

**Fig. 2:**
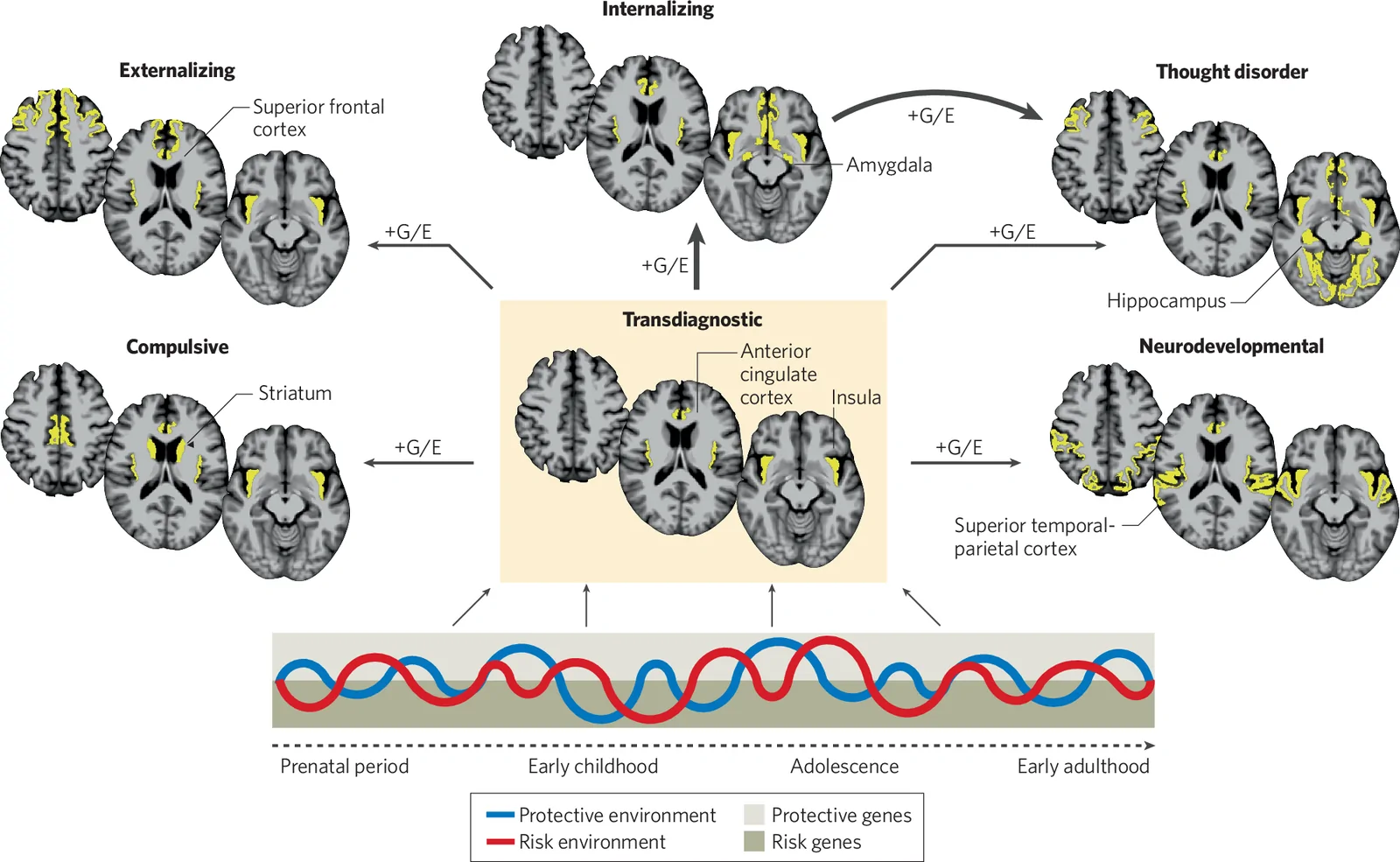
Proposed model of shared (transdiagnostic) and non-shared changes in the brain across five clusters of psychopathology. This model represents a hypothetical integration of: (1) the 5-factor genetics model generated by the recent analysis of genetic variants associated with 14 psychiatric disorders [49] and (2) the HiTOP model of the symptoms of psychiatric disorders [250], which include similar/overlapping clusters. (In this figure, the Substance Use Disorders factor of [49] is labeled the Externalizing factor, and the Schizophrenia/Bipolar Disorder factor is labeled the Thought Disorder factor, in order to be consistent with our emphasis on dimensional variation within and across these factors.) In this model, a shared Transdiagnostic factor is linked to changes in salience network regions (dorsal anterior cingulate cortex and insula). The salience network represents a plausible Transdiagnostic epicenter because it plays a central role in many core brain functions involved in affective processing and interactions between sensory, cognitive and affective processes, including: (1) perceiving and generating responses to salient stimuli in the environment, via influencing the activity of the autonomic nervous system and cognitive control systems; (2) top-down regulation of affective responses; (3) “switching” between internally and externally directed attention and other forms of goal-directed cognition; and (4) the integration of internal interoceptive signals with other types of sensory and cognitive information in the service of action selection [285, 286]. Certain genetic variants +/− their interactions with environmental events confer greater risk for progression of the Transdiagnostic factor-related neural vulnerabilities to involve other changes in the brain and symptoms (i.e., the 5 distinct factors), potentially via transynaptic propagation of functional impairment over long-range connections of the human brain. Note that some brain regions that may be components of vulnerable networks are not represented here (due to space constraints), including: the midbrain, hypothalamus, thalamus, and cerebellum, among others.

Extending these partially aligned models, we have constructed a novel hypothetical model (Fig. 2) that attempts to integrate these transdiagnostic genetic and psychopathology findings with findings of some of the cross-diagnostic neuroimaging meta-analyses described above. We also propose links to the “spreading pathology” hypothesis of neuropsychiatric illness [165, 167], as well as the clinical staging model [283, 284]. First, we hypothesize that there is a Transdiagnostic “epicenter” of vulnerability in the brain, potentially located in the salience network (the dACC and insula, and closely connected thalamic, hypothalamic, and midbrain areas), based on the findings of several transdiagnostic neuroimaging studies [151, 160, 282]. Second, we hypothesize that, in a subset of individuals, the pathology or dysfunction expressed in this core Transdiagnostic network is propagated over time to one or more additional closely-connected networks, and changes in these additional networks confer greater syndromal specificity. Therefore, a pathological process occurring within the Transdiagnostic epicenter network (hypothetically the salience network in this model), during childhood or adolescence, subsequently leads to changes in other networks, such as (most commonly) the Internalizing network (represented in this model by the ventromedial prefrontal cortex, amygdala, and ventral striatum).

In addition, in certain vulnerable individuals (due to specific genetic and/or environmental influences), the pathological process may further propagate to involve the Thought Disorder network (in this model represented by the hippocampus, lateral frontal areas, and sensory cortices) via one of two possible routes: (1) an indirect route, beginning in the Transdiagnostic network followed by involvement of the Internalizing network (given that, in the majority of cases (>60%) internalizing symptoms precede the onset of psychosis [253]) and then extending to involve the Thought Disorder network; or (2) a direct route, propagating from the Transdiagnostic network directly to the Thought Disorder network, which represents the pathway for individuals who develop psychosis with minimal preceding internalizing symptoms.

Lastly, other symptom clusters are hypothesized to arise via additional routes extending from the Transdiagnostic network, due to genetic and environmental influences and their interactions, which confer a specific vulnerability to changes in neural systems that give rise to those symptoms.

Overall, this theoretical model represents one potential integration of three different sources of transdiagnostic data. The goal of proposing this model is to provide one candidate framework for conceptualizing and further investigating how an array of diverse risk factors can generate, during the course of development, changes in brain and psychological function that result in shared and distinct forms of psychopathology. In addition, this model suggests several points of entry for interventions.

## Clinical implications and future directions

The model presented above has several testable implications for research and clinical care.

First, longitudinal studies can investigate whether a progression of psychopathology and associated changes in the brain occur over time in a subset of youth who have been identified as carrying elevated risk for such a progression, based on: (1) self-report or interview-based psychological, cognitive, or symptom screening instruments, and/or (2) objective behavioral tests or neurophysiological measurements. The influences of specific genetic and environmental risk and protective factors on these trajectories and the mental health outcomes; the degree to which such factors are common or distinct across mental health outcomes; and whether such outcomes are best characterized by a single and/or multiple cluster(s) of dimensionally-defined psychological traits or symptoms and their accompanying neurobehavioral profiles, can be comprehensively investigated.

Second, from a clinical perspective, if a common transdiagnostic psychological factor is primarily characterized by certain psychopathological dimensions (e.g., elevated neuroticism, emotional dysregulation and/or cognitive inflexibility), studies can test whether identifying any accompanying changes in the brain confers additional utility in terms of: (1) identifying youth who are particularly at risk and may benefit from additional evaluation, monitoring or intervention, and/or (2) tracking trajectories of illness and potential benefits of interventions over time. A direct comparison of screening approaches that do and do not incorporate objective measures of neurophysiology or behavior (following appropriate validation of such measures, establishing their reliability, and adequate predictive relationships with clinical progression) can begin to answer these questions. The rationale and need for validation and implementation of objective tests in psychiatry remains strong (despite the persistent elusiveness of this goal), given that self-report of symptoms can be influenced by impairments in judgment associated with the condition being evaluated, as well as demand characteristics of the test. However, it remains possible that many objective tests (e.g., behavioral or neuroimaging) will not provide additional benefit in terms of outcome prediction; existing validated clinical tools may be sufficient for many screening purposes.

**Fig. 3:**
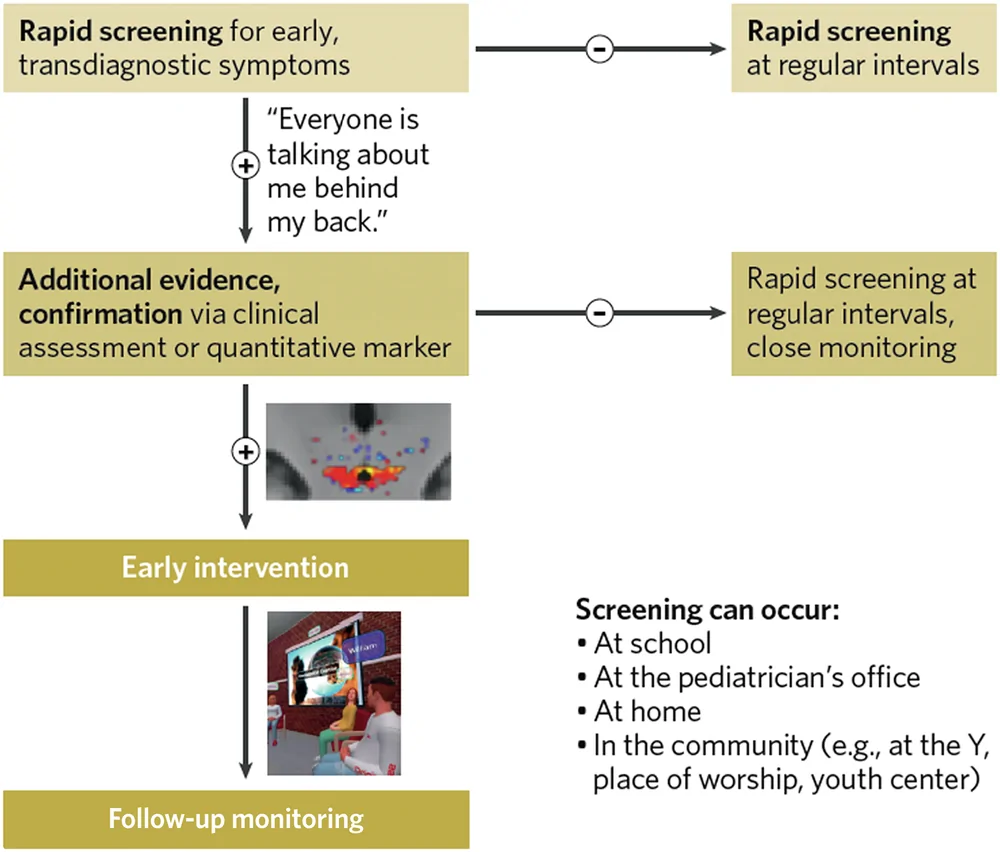
A candidate screening, early detection and prevention pathway of care. This diagram maps the sequential steps of a clinical screening and intervention algorithm that includes: (1) two sequential steps of screening, conducted using a symptom questionnaire followed by a clinical interview and/or an objective biomarker test; (2) repeat screening at regular intervals (e.g., annually) for those who screen negative; and (3) evaluation and intervention for those who test positive on both screening procedures. At the current time, the second screening test would likely consist of a clinical evaluation rather than an objective biomarker test, given that currently no such biomarkers have been sufficiently validated for this purpose (example hypothetical future biomarker included here: neuromelanin-sensitive MRI, a candidate marker of psychotic symptoms). The example intervention included here is the virtual reality-adapted version of Resilience Training, a four-session group intervention that aims to prevent worsening of transdiagnostic psychopathology in young adults [287–289].

Ideally, mental health screening occurs at several stages, using a variety of instruments. For example, certain screening measures could be used to identify individuals with some transdiagnostic risk (e.g., with elevated levels of a p factor, internalizing symptoms, psychotic experiences), and this type of screening may identify a large portion of the youth population. These mildly at-risk youth could then undergo further monitoring only, or participate in a low-burden, brief transdiagnostic intervention in addition. However, the predictive value of the self-reported marker of poor outcomes (and the cost-effectiveness of any recommended intervention) would likely increase if additional screening were conducted, either via: (1) a clinical interview or (2) a validated objective, behavioral, or biologically-based test. In Fig. 3, a schematic illustration of a screening, monitoring, and intervention pathway that includes these steps is presented. In this schematic pathway, a biologically-based screening test is included as one possible second-stage screener (versus a clinical interview). In this example, a neuromelanin-sensitive MRI scan is included as a candidate screening procedure that may, in the future, provide predictive information. This pathway illustrates one potential framework for translating research findings about clinical and biological markers of mental illness risk into a clinical screening and intervention pathway, which could be integrated with existing models of stage-based mental health care [283, 284].

In conclusion, psychiatry has lagged behind other fields of medicine in terms of progress towards implementing early detection and prevention-oriented approaches in the real world. This may be due, at least in part, to a continued reliance on categorical models of mental illness, which do not generally map onto what we have learned about the patterns of risk factors and early biological and psychological expressions of these conditions. Transdiagnostic models of mental illness risk and emerging psychopathology are now supported by convergent genetic, epidemiological, neurobiological and psychopathological findings. Therefore, at this point, such models should inform the further development and implementation of novel screening, assessment, and prevention-focused approaches beginning early in life, contributing to the ongoing efforts to address the mental health needs of youth and emerging adults in a cost-effective manner.
